# A directional total variation minimization algorithm for isotropic resolution in digital breast tomosynthesis

**Published:** 2024-06-11

**Authors:** Emil Y. Sidky, Xiangyi Wu, Xiaoyu Duan, Hailing Huang, Wei Zhao, Leo Y. Zhang, John Paul Phillips, Zheng Zhang, Buxin Chen, Dan Xia, Ingrid S. Reiser, Xiaochuan Pan

**Affiliations:** Department of Radiology MC-2026, The University of Chicago, 5841. S. Maryland Ave., Chicago, IL, 60637.; Department of Radiology, Renaissance School of Medicine, Stony Brook University, 101 Nicolls Road, Health Sciences Center, Level 4, Stony Brook, NY, 11794.; Department of Radiology, Renaissance School of Medicine, Stony Brook University, 101 Nicolls Road, Health Sciences Center, Level 4, Stony Brook, NY, 11794.; Department of Radiology, Renaissance School of Medicine, Stony Brook University, 101 Nicolls Road, Health Sciences Center, Level 4, Stony Brook, NY, 11794.; Department of Radiology, Renaissance School of Medicine, Stony Brook University, 101 Nicolls Road, Health Sciences Center, Level 4, Stony Brook, NY, 11794.; Department of Mathematics, University of Washington, Box 354350, C-138 Padelford, Seattle, WA 98195-4350.; Department of Radiology MC-2026, The University of Chicago, 5841. S. Maryland Ave., Chicago, IL, 60637.; Department of Radiology MC-2026, The University of Chicago, 5841. S. Maryland Ave., Chicago, IL, 60637.; Department of Radiology MC-2026, The University of Chicago, 5841. S. Maryland Ave., Chicago, IL, 60637.; Department of Radiology MC-2026, The University of Chicago, 5841. S. Maryland Ave., Chicago, IL, 60637.; Department of Radiology MC-2026, The University of Chicago, 5841. S. Maryland Ave., Chicago, IL, 60637.; Department of Radiology MC-2026, The University of Chicago, 5841. S. Maryland Ave., Chicago, IL, 60637.

**Keywords:** Limited angular-range reconstruction, digital breast tomosynthesis, dual-energy, contrast-agent imaging

## Abstract

An optimization-based image reconstruction algorithm is developed for contrast enhanced digital breast tomosynthesis (DBT) using dual-energy scanning. The algorithm minimizes directional total variation (TV) with a data discrepancy and non-negativity constraints. Iodinated contrast agent (ICA) imaging is performed by reconstructing images from dual-energy DBT data followed by weighted subtraction. Physical DBT data is acquired with a Siemens Mammomat scanner of a structured breast phantom with ICA inserts. Results are shown for both directional TV minimization and filtered back-projection for reference. It is seen that directional TV is able to substantially reduce depth blur for the ICA objects.

## I. Introduction

Over the past two decades, X-ray based tomosynthesis has been an emerging imaging modality that has been making great strides in clinical adoption; most notably, digital breast tomosynthesis (DBT) is becoming the workhorse imaging device for breast cancer screening. Although DBT yields partially tomographic volumes, there is still a large gap between it and X-ray computed tomography in terms of achieving isotropically high resolution and in the ability to achieve quantitive imaging for shape and volume features of tissues within the scanned subject. Tomosynthesis imaging is subject to significant depth blur, and this blurring is proportional to the size of the imaged structures. Because of this depth blurring it is not possible to outline the boundaries of structures and estimate their volume. Moreover, the gray scale values in the image do not directly correspond to a physical density.

In this work, an optimization-based algorithm is developed that may enable quantitive imaging with limited angular range scanning such as what is performed in a tomosynthesis acquisition. In order to accomplish this, we exploit sparsity in the gradient magnitude of the scanned subject. For sparse-view imaging over a full scanning angular range, the use of total variation (TV) minimization has proven effective [1]. For limited angular range scanning, however, directional TV constraints show promise [2]. The use of directional TV constraints can be shown to be equivalent to directional TV minimization where the gradient magnitudes in different directions have different penalty weights [3]. The work presented in Ref. [3] showed only simulation studies, and in this work we apply this algorithm to physical DBT transmission data.

The application of interest is contrast enhanced (CE) DBT [4], where the goal is to obtain a quantitative image of the Iodinated contrast agent (ICA) distribution. If such a system is successfully developed it could enable the use of CE-DBT in assessment of breast cancer therapy effectiveness. For this preliminary work, DBT data is obtained for structured breast phantom with ICA inserts. Volumes are reconstructed from scans at 30 and 49 kV, and the weighted image subtraction method is used to isolate the ICA distribution.

## II. Methods

### A. Image reconstruction

In order to obtain reconstructed DBT volumes that have nearly isotropic resolution for the ICA distribution, we exploit the fact that this distribution is localized with a fairly simple structure and high contrast. In the algorithm implementation directional gradient sparsity regularization is employed and the resolution is reduced by a factor of eight from the native resolution of the DBT system’s detector.

The image reconstruction algorithm is developed for the Siemens Mammomat scanner that acquires 25 projections over a 50 degree scanning arc and the detector resolution is 85 microns. The transmission data are processed with the negative logarithm after dividing by a flood-field scan and the relationship between this processed data and the DBT volume is assumed to be standard linear X-ray projection as described by

g=Xf,

where f is a discrete representation of the DBT volume, X is a matrix encoding X-ray projection, and g is the processed projection data.

For sparsity regularization, image reconstruction is formulated using an optimization model where weighted image directional total variation (TV) is minimized subject to a data-fidelity constraint. Furthermore, an $\ell_{1}$ penalty term is included that encourages pixel sparsity and helps to confine the image to the true object support. Finally, the data fidelity term compares ramp-filtered data with ramp-filtered estimated data, which has two important effects: this filtering deemphasizes low spatial-frequency discrepancy in the sinogram estimation and it also performs preconditioning.


$$\begin{array}{ll} f=\underset{f^{'}}{arg\;min}\left\{\alpha {∥\nabla_{x}f^{'}∥}_{1}+\alpha {∥\nabla_{y}f^{'}∥}_{1}+(2-\alpha )∥\nabla_{z}f^{'}∥\right.\\ & \left.+\beta {∥f^{'}∥}_{1}\;\text{such}\;\text{that}\;{∥R\left(g-Xf^{'}\right)∥}_{2}\leq \epsilon \;\text{and}\;f^{'}\geq 0\right\}. \end{array}$$

The x and y coordinates are in-plane with y being along the direction of travel for the X-ray source, and the z direction is the depth direction; i.e. perpendicular to the X-ray detector. The symbol ∇ represents finite differencing along the direction indicated by the subscript; α is a weighting parameter that should be chosen between 0 and 2; β is the weighting parameter for the $\ell_{1}$ penalty, R represents ramp filtering and ϵ is a data error tolerance parameter. The posed optimization problem is convex and it can be solved with the primal-dual algorithm of Chambolle and Pock [5].

When α=1, the gradient sparsity regularization term is equivalent to TV and it has been shown that constrained TV minimization is effective for accurate image reconstruction for sparse-view acquisition when the scanning arc is complete. Recently, we have shown that the anisotropic weighting of the directional TV terms can improve image reconstruction accuracy for limited scanning angular ranges such as those used for DBT. This in combination with use of a coarse volume grid allows for nearly isotropic resolution in the reconstructed DBT images using directional TV minimization.

### B. Image post-processing

Even when the transmission data preprocessing accurately estimates the image sinogram, Reconstructed DBT images are susceptible to low spatial-frequency artifact due to the limited angular range of the scan. In order to facilitate the dual-energy image subtraction the low spatial-frequency artifacts are estimated and removed by normalization. Specifically, the low spatial-frequency image component is estimated by robust polynomial fitting

(1) $$b=\underset{c}{arg\;min}∥m\cdot \left(s-Pc\right){∥}_{1},$$

where s is an in-plane slice of the image f;P is a matrix of low-order polynomials; c is a vector of coefficients; m is an indicator function which is one on the object support and zero otherwise, where the object support is estimated by thresholding the image slice, s; and b is the estimated background for the slice *s*. The use of the $\ell_{1}$-norm for the objective function encourages the difference image in its argument to be sparse, and this design should preserve important small structures in the image when the background drift is processed away.

The polynomial fitting is performed slice by slice with the result stacked into the background image $f_{b}$. Likewise, the mask image $f_{m}$ is assembled by stacking the slice masks. The algorithm for solving this robust polynomial fitting is iteratively reweighted least-squares. The final displayed image, $f_{d}$, is computed as follows

(2) $$f_{d}=f_{m}\cdot \frac{f}{f_{b}}.$$

After the processed images are computed for low and high kV, a difference image is computed

$$f^{\left(ICA\right)}=\left(f_{d}^{\left(HE\right)}-1\right)-w\cdot \left(f_{d}^{\left(LE\right)}-1\right).$$

For this work, the dual energy data is processed in the image domain by performing a weighted subtraction of low and high kV reconstructed volumes, where the weights are designed to highlight the ICA distribution. Because the low spatial-frequency background variation is reduced by normalization, the background of the processed images center on the value of 1; thus 1 is subtracted from both images before performing the weighted subtraction. The weighting parameter *w* is selected by visual inspection.

### C. DE-DBT transmission data acquisition

**Fig. 1.**
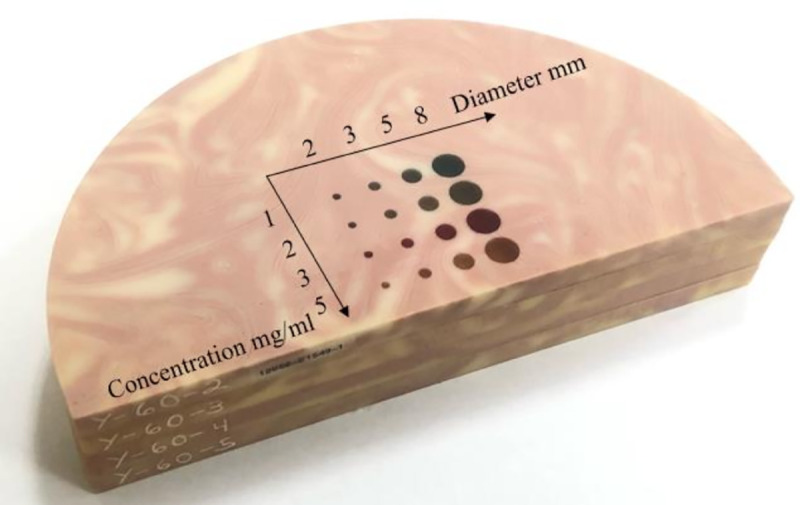
CIRS phantom with solid ICA inserts. The background is hetergeneous with 50% glandularity.

The DE-DBT scanning consists of two DBT acquisitions taken at source potential of 30 and 49 kV using filtration with Al and Ti, respectively. DE-DBT scans of a 4 cm thick BR3D phantom (Model 020, CIRS Inc. Norfolk, VA) with solid ICA inserts, see Fig. 1, are used for testing the proposed image reconstruction algorithm. The ICA inserts vary in size and concentration; the diameters are 2, 3, 5, and 8 mm and the Iodine concentrations are 1, 2, 3, and 5 mg/mL.

## III. Results

For the present implementation of directional TV minimization, the weighting parameter α is set to 1.75, favoring the in-plane differentiation in the objective function. The $\ell_{1}$ penalty parameter β is set to 0.1, and the data error constraint ϵ is 0.01 in terms of root mean square error. The imaging volume is discretized on a coarse grid of size 188 × 417 × 175 with cubic voxels 680 microns wide.

**Fig. 2.**
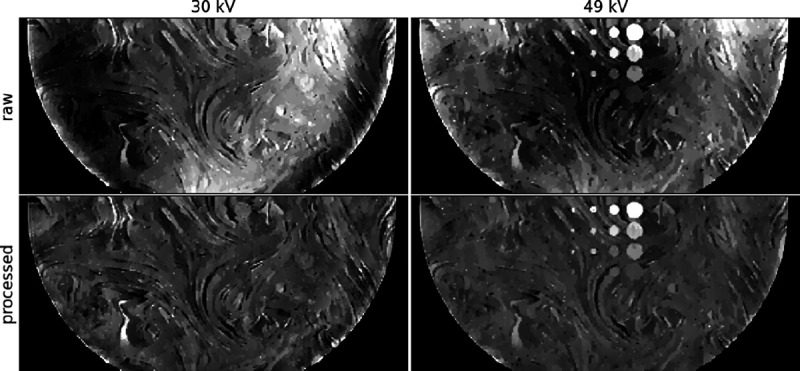
Raw and processed in-plane images containing all ICA objects for the directional TV minimization images for the 30 kV (Left) and 49 kV (Right) DBT scans. Image processing is performed by polynomial fitting and normalization described in Eqs. (1) and (2), respectively. The gray scale for the raw images is selected to span the range of background variation, and the gray scale for the normalized images is selected to be ± 30% of background.

Raw slice images from applying directional TV minimization to the DBT phantom data are shown in the top panels of Fig. 2, and the low spatial-frequency artifacts are clearly visible. The slice shown cuts through all of the ICA objects. These objects are easily seen in the 49 kV images, and not the 30kV images, because the K-edge of Iodine is 33.2 keV. The spatial dependence of the artifact is different for the low and high kV images, and therefore direct image subtraction of the raw images will not yield good isolation of the ICA distribution. For performing the post-processing, we employed an eighth degree 2D polynomial expansion (45 terms), and resulting images are shown in the bottom row of Fig. 2, where it is seen that the low spatial-frequency artifacts are greatly reduced.

**Fig. 3.**
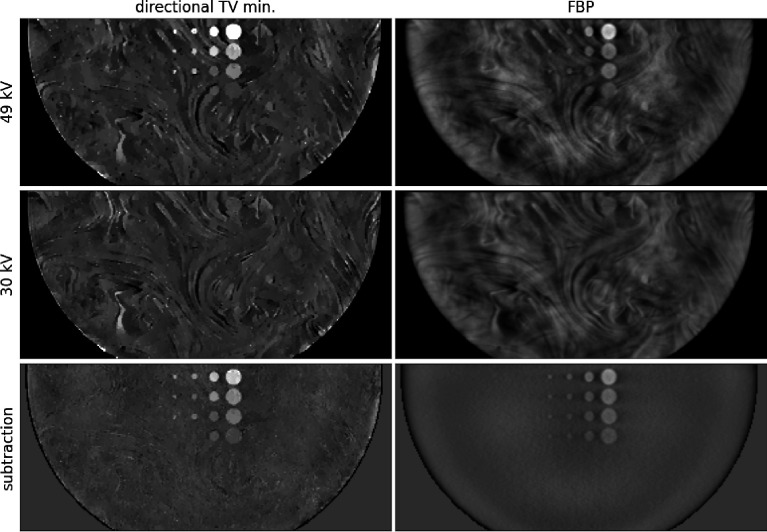
In-plane images containing all ICA objects for directional TV minimization (Left) and FBP (Right). The top and middle rows show the images corresponding to the 49 and 30 kV acquisitions, respectively. The bottom row shows the weighted subtraction designed to isolate the ICA distributions. The gray scales on the left and right are matched and the images are masked to the object support.

The results of weighted subtraction for directional TV minimization filtered back-projection (FBP) are shown in Fig. 3, where it is seen in the bottom row that it effectively suppresses the background variation and isolates the ICA objects in the image.

**Fig. 4.**
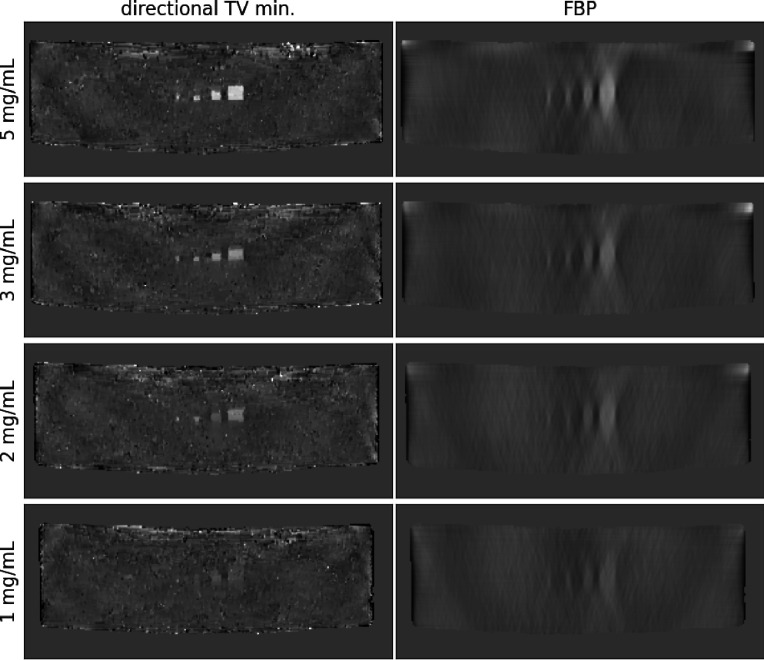
Transverse plane images cutting through the groups of ICA objects of the same concentration with directional TV minimization and FBP images on the left and right, respectively. Concentration of the ICA decreases going from top to bottom. All gray scales are matched for quantitative comparison and the images are masked to the object support.

Transverse slices, where y is horizontal and z is vertical, for the weighted subtraction of directional TV minimization and FBP images are displayed in Fig. 4. While the FBP images show the usual depth blurring of the ICA objects, it is clear that directional TV minimization can effectively mitigate this artifact. On the other hand, the directional TV difference images have slightly stronger background clutter due to imperfect cancellation of the background glandular structures, which also interferes with the imaging of the 1mg/mL ICA inserts. This imperfect cancellation is likely due to the non-linearity of the sparsity regularization.

## IV. Conclusion

In this preliminary study on image reconstruction by directional TV minimization for CE-DBT, the physical phantom results show promise in achieving an accurate ICA distribution estimate with nearly isotropic spatial resolution; it appears that the depth blurring artifact common to DBT imaging is well controlled with directional TV minimization. Because the dual-energy processing, in this work, is image-based the gray values in the ICA images are not quantitive and cannot be directly tied to a concentration value. Future work will focus on spectral calibration of the DBT transmission data so that both one-step and two-step [6] quantitative DE-DBT image reconstruction can be developed. Adapting directional TV minimization to a quantitive DE-DBT is expected to greatly reduce background clutter in the ICA images from glandular tissue, and this should also provide accurate ICA concentrations.
